# Regionalization and regulation: impact on access to cancer treatment

**DOI:** 10.11606/s1518-8787.2026060007257

**Published:** 2026-03-16

**Authors:** Luciane Simões Duarte, Mirian Matsura Shirassu, Adeylson Guimarães Ribeiro, Cristiane Murta-Nascimento, Marcela de Araújo Fagundes, Carolina Terra de Moraes Luizaga, Victor Wünsch

**Affiliations:** I Universidade de São Paulo. Faculdade de Saúde Pública. São Paulo, SP, Brasil; II Secretaria de Estado da Saúde de São Paulo. Centro de Vigilância Epidemiológica, Divisão de Doenças Crônicas Não Transmissíveis. São Paulo, SP, Brasil; III Fundação Oncocentro. São Paulo, SP, Brasil; IV Universidade Estadual Paulista. Faculdade de Medicina. Botucatu, SP, Brasil

**Keywords:** Medical Oncology, Regional Health Planning, Health Services Accessibility, Cancer Care Facilities

## Abstract

**OBJECTIVE:**

To assess the impact of Redes Regionais de Atenção à Saúde (RRAS - Regional Health Care Network) and Oncology Regulation on geographical accessibility to oncology treatment for the main types of cancer in the state of São Paulo.

**METHODS:**

A before-after study comparing the three-year periods before (2007–2009) and after (2017–2019) the policies were implemented. Data from the São Paulo Hospital Cancer Registry were used to analyze indicators of displacement and attraction of patients for surgery, radiotherapy, or chemotherapy in six types of cancer: stomach, colorectal, lung, female breast, cervix, and prostate. The RRAS were adopted as the geographical units of analysis, and inter-RRAS displacements were categorized into quartiles (< 25%, 25%–49%, 50%–74%, and ≥ 75%). Temporal analysis was carried out using thematic maps (QGIS, version 3.28) and the chi-squared test (α = 5%).

**RESULTS:**

Approximately 25% of patients traveled for treatment. RRAS 6 (Capital), 9 (Bauru), and 13 (Ribeirão Preto) remained the centers of attraction. In the North and Northwest, RRAS 11 and 12 showed a reduction in displacements for all types of cancer; in RRAS 10, the drop occurred for colorectal and lung tumors. In the Southeast, RRAS 3 and 5 do not have qualified services, requiring displacements, while RRAS 1, 2, and 4 maintained high percentages. In the East, RRAS 14 to 17 saw a reduction in displacements, except for lung cancer. In the South, displacements increased in RRAS 7 and 8. Overall, there was a reduction in displacements for cervical and prostate cancer, and an increase for lung cancer, especially for surgery.

**CONCLUSION:**

The implementation of the RRAS and Oncology Regulation altered geographic accessibility in a heterogeneous way: there was an improvement in the North and Northwest, a worsening in the South, and stability in the reference centers. There is a need to improve regional planning, paying attention to territorial inequalities, the type of tumor, and the therapeutic modality.

## INTRODUCTION

In 2023, 125,200 new cases of cancer were estimated in the state of São Paulo (excluding non-melanoma skin cancer), 51.3% of which were in women. The most common types were: breast, prostate, colorectal, trachea, bronchus and lung, stomach, and cervix^1^.

In Brazil, cancer care for diagnosis and treatment is organized into three main types of specialized services: the Unidades de Assistência de Alta Complexidade em Oncologia (Unacon - High Complexity Oncology Care Units), which treat the most prevalent types of cancer; the Centros de Assistência de Alta Complexidade em Oncologia (Cacon - High Complexity Oncology Care Centers), which offer treatment for all types of cancer, except, in some cases, rare or pediatric tumors; and the general hospitals, which perform cancer surgeries and, when necessary, refer patients for complementary therapies^2^.

The Ministry of Health has implemented two initiatives with the aim of improving the organization and flow of cancer patients in the Unified Health System (SUS). The first, in 2008, was the introduction of Regulation in the SUS, with the aim of structuring the supply of services, guaranteeing fair access and ensuring comprehensive care^3^. The second initiative, in 2010, was the creation of Redes de Atenção à Saúde (RAS - Health Care Networks), designed to reduce the fragmentation of the system, increase accessibility and integrate services of different levels of complexity, with the support of logistical and management systems^4,5^.

In the state of São Paulo, these policies resulted, in 2010, in the creation of the Central de Regulação de Ofertas de Serviços de Saúde (Cross - Health Services Offer Regulation Center) and, later, in the implementation of Oncology Regulation^6^. In 2012, the Redes Regionais de Atenção à Saúde (RRAS - Regional Health Care Networks) were established, with the aim of organizing services in a regionalized and integrated manner^7^.

Despite the progress made in organizing the system, geographical accessibility to cancer treatment is still a major challenge. National data shows that between 49.2% and 60.7% of cancer patients need to travel from their municipalities of residence to receive specialized care^8^. This situation is the result of the planned centralization of cancer services, which does not provide treatment in all municipalities. Although this guideline aims to guarantee quality and comprehensive care, it entails additional financial and emotional costs for patients and their families^13^.

The concept of accessibility goes beyond simple access to health services, as it encompasses both the user’s entry into the system and the capacity to provide care and respond to the population’s health needs. Accessibility can be analyzed in two dimensions: socio-organizational, which covers the characteristics of the services on offer, except for those of a geographical nature, and geographical, which considers the spatial aspects that can represent barriers to the user’s movement. The latter can be measured using indicators such as linear distance, travel time and cost, among others^14^.

The aim of this study was to assess the impact of RRAS and Oncology Regulation on geographical accessibility to cancer treatment for the main types of cancer in the state of São Paulo, helping to fill gaps in knowledge about the evolution of geographical accessibility to cancer treatment in the SUS and the effects of specific policies implemented in this area.

## METHODS

This before-after study compared two independent three-year periods: pre-intervention (2007–2009), before the implementation of the RRAS and Oncology Regulation in the state of São Paulo, and post-intervention (2017–2019), after the consolidation of these strategies. The use of three-year periods reduces the influence of one-off variations in patient displacement, contributing to the stability of the indicators.

### Characterization of the Study Area

Despite occupying only 2.9% of Brazil’s territory, the state of São Paulo concentrates 21.9% of the country’s population, i.e. more than 44 million inhabitants spread over 645 municipalities^15^. In this analysis, the RRAS were adopted as the geographical reference units. Although this regionalization was not yet in force in the 2007–2009 triennium, it was decided to use it in both periods to ensure comparability of results. The RRAS are not limited to geographical or administrative divisions, but reflect epidemiological, demographic, and socioeconomic characteristics and the organization of the local health services network. In 2024, the São Paulo State Health Department promoted a reorganization, increasing the number of RRAS to 18^16^. In this study, however, the division into 17 RRAS was maintained, according to the configuration in force during the two periods analyzed.

### Database

We used data from the Registro Hospitalar de Câncer de São Paulo (RHC/SP - São Paulo Cancer Hospital Registry), a state system managed by the Fundação Oncocentro de São Paulo (FOSP - São Paulo Oncocenter Foundation), which gathers information on cases treated in highly complex oncology establishments^17^.

In the two three-year periods analyzed, 66 institutions registered cancer cases in the RHC/SP, 48 of which were Unacon, 15 Cacon, and three general hospitals. Although the total number of services remained stable between the periods, there were changes in two RRAS: in RRAS 6, there was a reduction from six to five Unacons; and in RRAS 8, there was an increase from two to three Unacons.

### Inclusion Criteria

Patients ≥ 20 years old, living in the state of São Paulo, diagnosed with cancer during the study periods and according to the International Classification of Diseases for Oncology (ICD-O-3)^18^ for stomach (C16), colorectal (C18 to C20), bronchus and lung (C34), female breast (C50), cervix (C53), or prostate (C61) cancer, including *in situ* cases, undergoing at least one of the treatments, surgery, radiotherapy, or chemotherapy, were included. The final sample comprised 52,128 patients between 2007 and 2009 and 69,750 patients between 2017 and 2019.

### Study Variables

Geographical accessibility was assessed by two indicators adapted from Dama et al.^19^:

- Patient displacement:

$$\frac{\text{Number of patients from the RRAS of residence who underwent treatment at another RRAS}}{\text{Total number of patients from the RRAS of residence who underwent cancer treatment }X100)}$$

- Patient attraction:

$$\frac{\text{Number of non-resident patients whounderwent treatment at RRAS}}{\text{Total number of treatments performed at RRAS}X100}$$

The triennium of diagnosis, primary tumor location, tumor staging according to the TNM system^20^, and therapeutic modality (surgery, radiotherapy, or chemotherapy) were analyzed. Moreover, the sociodemographic characteristics of the patients were examined, including gender (male or female), education level (illiterate, complete or incomplete primary education, secondary education, or higher education), and age (in years).

### Data Analysis

Inter-RRAS displacements were categorized into quartiles (< 25%, 25%–49%, 50%–74%, and ≥ 75%), for ease of interpretation and to generate more accessible and intuitive scientific evidence. The temporal analysis was carried out using thematic maps comparing the periods (QGIS, version 3.28) and the chi-square test (α = 5%). R software (version 4.2.0) was used for the analysis procedures.

### Ethical Aspects

The study was approved by the Research Ethics Committee (CAAE: 75425623.0.0000.5421), in accordance with the guidelines of Resolutions 466/12 and 510/16 of the National Health Council.

## RESULTS

Between the periods 2007–2009 and 2017–2019, there was an increase in the number of registrations at the RHC/SP for all the types of cancer analyzed. Colorectal cancer showed the highest percentage increase (58.2%), from 8,159 to 12,906 cases. This was followed by breast cancer, with an increase of 46.3% (from 16,413 to 24,007). Lung, cervical, and prostate tumors increased by almost 20.0% each: from 4,748 to 5,787, from 5,755 to 6,845, and from 12,932 to 15,920 cases, respectively. Stomach cancer had the smallest variation, with an increase of just 3.4% (4,121 to 4,285).

There was a similar profile among the patients who moved in the two three-year periods analyzed, with close proportions of men and women moving for cancer treatment (around 25%) and an average age of 58. Between the first and second three-year periods, there was a reduction in the number of people with no schooling (from 30.8% to 24.4%) and an increase among those with primary schooling (from 19.0% to 21.0%). The frequencies of early (TNM I and II) and advanced (TNM III and IV) tumor stages by RRAS were also similar between the periods. However, among individuals from RRAS 4, 6, 12, 14, and 16 with tumors in early stages, there was an increase in displacement, while RRAS 13 and 15 showed a decrease. Among patients with advanced tumors, there was a reduction in displacement in RRAS 9, 13, and 15.

Around a quarter of cancer patients in the state of São Paulo had to travel outside their RRAS of residence for treatment. Cervical cancer was the only one to show an overall reduction in displacements for all therapeutic modalities. Prostate cancer saw a reduction in total displacement. Lung cancer, on the other hand, saw an increase, especially in surgeries (+13.6%). Stomach, colorectal, and breast tumors maintained stable total displacements (Table 1).

**Table 1:** Distribution of cancer patients with inter-RRAS displacement for treatment, by type of cancer, therapeutic modality, and three-year period. State of São Paulo, 2007–2009 and 2017–2019.

Type of cancer and treatment	2007–2009		2017–2019		Percentage change	p^a^
	n	%	n	%		
Stomach						
Surgery	858	28.8	726	29.7	+3.1	0.489
Radiotherapy	260	29.2	168	26.4	-9.6	0.254
Chemotherapy	669	27.7	870	29.3	+5.8	0.228
Total	1,787	28.5	1,764	29.1	+2.4	0.411
Colorectal						
Surgery	1,606	25.0	2,228	24.4	-2.4	0.347
Radiotherapy	598	26.1	796	28.2	+8.0	0.106
Chemotherapy	1,245	22.1	1,802	21.6	-2.3	0.498
Total	3,449	24.1	4,826	23.8	-1.2	0.535
Lung						
Surgery	234	21.4	328	24.3	+13.6	0.097
Radiotherapy	592	28.4	647	27.2	-4.2	0.382
Chemotherapy	949	25.5	1,130	27.0	+5.9	< 0.001
Total	2,071	23.5	2,105	26.6	+11.5	< 0.001
Breast						
Surgery	3,338	24.9	4,697	26.3	+5.6	0.007
Radiotherapy	2,189	25.7	2,704	24.5	-4.7	0.069
Chemotherapy	2,770	24.8	3,609	23.8	-4.0	0.078
Total	8,297	25.1	11,010	25.0	-0.3	0.841
Cervix						
Surgery	1,304	32.4	1,243	28.0	-13.6	< 0.001
Radiotherapy	625	28.4	543	24.7	-13.0	0.005
Chemotherapy	359	24.3	548	23.5	-3.3	0.573
Total	2,288	29.7	2,334	26.0	-14.3	< 0.001
Prostate						
Surgery	2,044	32.9	2,309	34.0	+3.3	0.171
Radiotherapy	1,081	23.1	1,092	19.0	-17.7	< 0.001
Chemotherapy	218	17.2	191	11.1	-35.5	< 0.001
Total	3,343	27.5	3,592	25.2	-9.1	< 0.001

RRAS: Redes Regionais de Atenção à Saúde (Regional Health Care Networks).

^a^Chi-square test.

**Figure 1:**
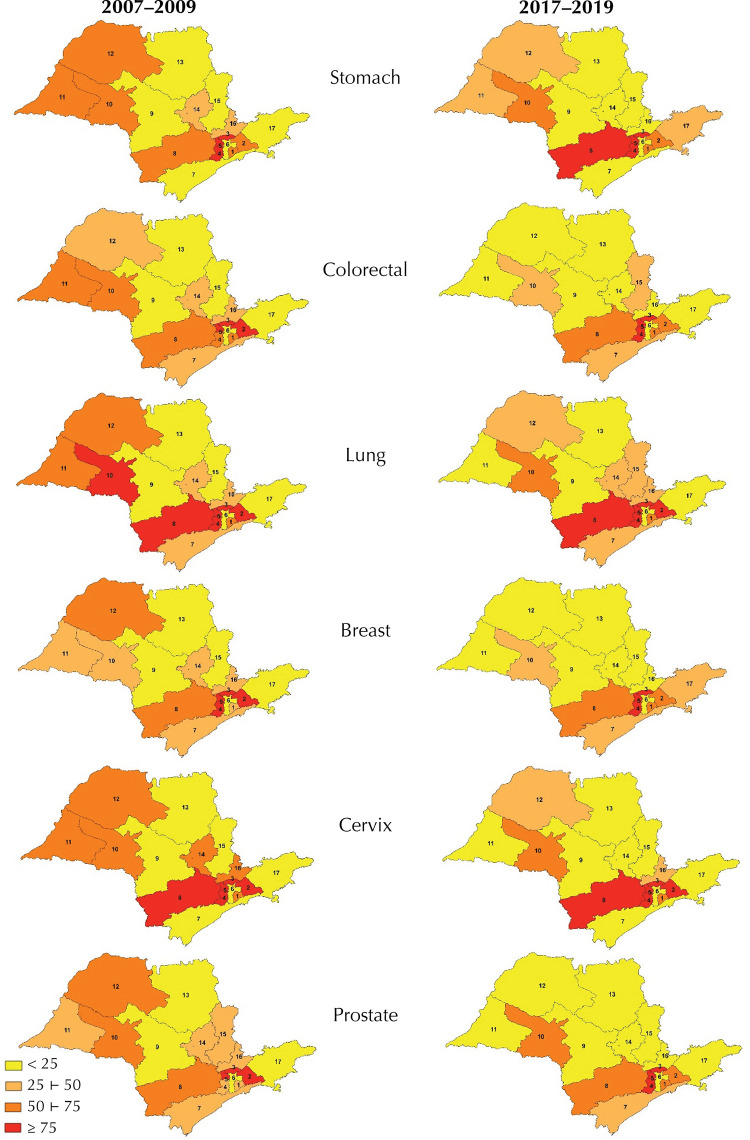
Spatial distribution of RRAS by quartile of patient displacement, by type of cancer and three-year period. State of São Paulo, 2007–2009 and 2017–2019.

Figure 1 shows the variations in quartiles of patient movements between RRAS, by type of cancer and three-year period. The mapping reveals distinct regional patterns of mobility for cancer treatment. In the Northwest region (RRAS 10, 11, and 12), there was a reduction in displacements for all tumors in RRAS 11 and 12. The North and Central regions (RRAS 9 and 13) maintained low displacement percentages, indicating relative self-sufficiency in cancer care. In the East region (RRAS 14 to 17), there were heterogeneous variations in displacement, depending on the type of cancer. In the South (RRAS 7 and 8), the percentage of displacements remained stable, except for RRAS 8. In the Southeast (RRAS 1 to 5), RRAS 3 and 5 stand out, as they do not have establishments qualified in high-complexity oncology, resulting in compulsory displacements. Furthermore, RRAS 6 maintained low percentages of displacement, indicating good installed capacity and local resolution.

**Figure 2:**
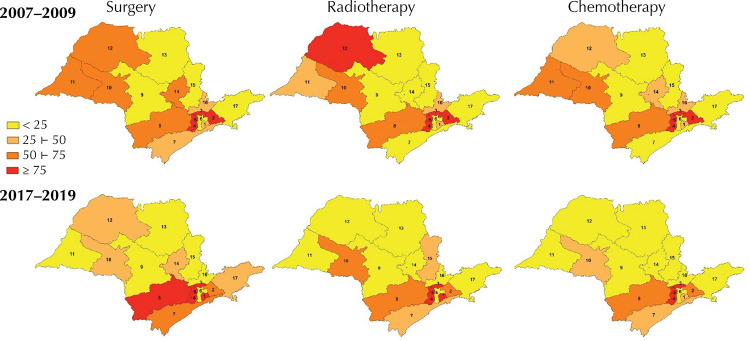
Spatial distribution of RRAS by quartile of patient displacement, by therapeutic modality and three-year period. State of São Paulo, 2007–2009 and 2017–2019.

Figure 2 shows the variations in patient displacement between the RRAS, according to therapeutic modality. In the Northwest region (RRAS 10, 11, and 12), there were reductions in displacements by modality; while in the South of the state (RRAS 7 and 8), there was an increase in displacements. In the Southeast (RRAS 1 to 5), the RRAS maintained high percentages of displacement, except for RRAS 2, which showed a reduction for all therapeutic modalities.

Table 2 shows the proportions of patients attracted by RRAS in the two three-year periods. Three RRAS - 6, 9 and 13 - stood out as reference centers, receiving more than 25% of cases from other regions, in all therapeutic modalities. RRAS 6 covers the capital, and RRAS 9 and 13 bring together populous municipalities with good health infrastructure. RRAS 9 includes Bauru, Botucatu, and Jaú, and RRAS 13 includes Ribeirão Preto, Franca, São Carlos, Araraquara, and Barretos - all with more than 100,000 inhabitants. These RRAS showed different trends in attracting patients. RRAS 6 saw an increase in the volume of cases referred to from other regions, but RRAS 9 and 13 saw a reduction in this flow.

**Table 2:** Proportion of cancer patients attracted by RRAS, by therapeutic modality and three-year period. State of São Paulo, 2007–2009 and 2017–2019.

RRAS of attraction	2007–2009			2017–2019		
	Surgery	Radiotherapy	Chemotherapy	Surgery	Radiotherapy	Chemotherapy
1	8.5	6.7	8.1	12.4	1.3	6.1
2	1.1	0.0	1.8	8.1	5.8	7.4
3	-	-	-	-	-	-
4	14.0	3.8	10.3	29.1	0.0	0.0
5	-	-	-	-	-	-
6	28.0	27.9	28.4	34.9	32.6	35.7
7	0.0	0.0	0.3	0.6	0.3	0.6
8	0.9	0.3	1.1	0.3	0.3	0.4
9	52.7	50.7	49.4	42.4	41.1	41.7
10	0.8	1.0	0.4	0.6	1.4	0.6
11	0.4	0.6	0.5	1.2	1.4	0.5
12	0.9	0.0	0.7	2.3	0.8	1.0
13	39.6	37.8	38.0	28.8	26.2	27.3
14	2.6	9.6	3.3	1.9	16.2	2.5
15	16.1	14.0	12.8	6.3	5.6	4.9
16	4.8	7.4	7.2	11.5	14.5	13.6
17	0.2	0.2	0.2	0.0	0.0	0.0

RRAS: Redes Regionais de Atenção à Saúde (Regional Health Care Networks).

Although they treat patients from all over the state, most cases attracted by these three RRAS remained concentrated in adjacent regions, a pattern observed in both three-year periods (Table 3).

**Table 3:** Proportion of non-resident patients treated in the three main RRAS of attraction, by RRAS of origin, therapeutic modality, and three-year period. State of São Paulo, 2007–2009 and 2017–2019.

Type of treatment and RRAS of attraction	2007–2009		2017–2019	
	RRAS of origin	% of patients from the RRAS of origin	RRAS of origin	% of patients from RRAS of origin
RRAS of attraction 6				
Surgery	2	23.5	5	22.9
	5	22.6	2	20.0
	1	19.9	1	18.6
Radiotherapy	2	24.1	5	25.9
	5	23.1	1	20.2
	1	18.8	2	17.3
Chemotherapy	2	23.8	5	26.1
	5	23.5	2	19.3
	1	18.3	1	17.0
RRAS of attraction 9				
Surgery	10	28.8	8	44.4
	8	25.1	10	33.5
	13	14.4	13	12.2
Radiotherapy	10	29.4	8	49.1
	8	26.6	10	28.7
	14	13.2	13	11.2
Chemotherapy	10	31.7	8	45.1
	8	24.7	10	34.2
	14	14.5	13	9.0
RRAS of attraction 13				
Surgery	12	80.1	12	69.6
	15	6.5	15	10.0
	11	2.5	8	3.9
Radiotherapy	12	80.0	12	57.5
	15	6.9	15	11.6
	11	2.8	8	6.6
Chemotherapy	12	78.3	12	54.8
	15	7.7	15	12.7
	11	2.9	8	6.1

RRAS: Redes Regionais de Atenção à Saúde (Regional Health Care Networks).

## DISCUSSION

The results showed significant changes in geographical accessibility to oncology services in the state of São Paulo after the implementation of the RRAS and Oncology Regulation. There was an increase in patients with lung cancer traveling outside their RRAS of residence in search of treatment, while cases of cervical and prostate cancer showed a reduction in displacement.

The North and Northeast regions of the state showed a reduction in travel for all types of cancer treatment. In contrast, the South saw an increase in these displacements, especially for surgical procedures. The percentage of trips from neighboring regions to the capital remained high, showing inequalities in the distribution of cancer care services in the Metropolitan Region of São Paulo.

The data from this study show significant inequality in access to cancer care in the state of São Paulo, reflecting persistent challenges, even after the organization of the RRAS and Oncology Regulation. There is a concentration of services in specific centers, such as RRAS 6 (São Paulo - Capital) and RRAS 13 (Barretos and Ribeirão Preto region), which concentrate large reference centers with a high capacity to attract patients. On the other hand, the scarcity of high-complexity services in regions such as RRAS 3 (Franco da Rocha) and RRAS 5 (Rota dos Bandeirantes) forces residents to travel to other territories in search of care.

The study also showed that even in RRASs with qualified oncology services, notably RRAS 1 (Grande ABC), 2 (Alto Tietê), 4 (Mananciais), 8 (Sorocaba region), and 10 (Marília region), the percentage of population displacement exceeded the state average. The greatest vulnerabilities in cancer care in the state are concentrated in the regions adjacent to the capital and in the south and southeast.

The displacement of cancer patients has been widely documented by studies carried out in Brazil. In the state of Rio de Janeiro, a study based on social network analysis showed significant differences in the flow of care and displacement of patients with digestive tract cancer, highlighting the persistent centrality of the capital^11^. In São Paulo, a study carried out at a Unacon located in São Bernardo do Campo found that 43% of patients came from other states and revealed inconsistencies in address records in 10% of medical records^9^. Nationwide, an analysis of more than 12 million cancer procedures found that patients from the North and Central West regions travel the greatest distances for treatment due to the concentration of cancer care centers in the Southeast and Northeast regions^12^. These disparities continue over time, highlighting the need to review the distribution of specialized oncology services in the country.

In addition to the presence of qualified services, it is important to consider the number and type of oncology services, as these factors allow us to identify care gaps in the state. RRAS 1, 2, 4, 8, 10, 11, 12, 16, and 17, although they all have Unacon and, in some cases (RRAS 7, 10, and 12), also Cacon, showed high percentages of displacement. On the other hand, as expected, RRASs with a large number of qualified services (6, 9, and 13) stood out as centers of attraction. However, RRAS 14 and 15, despite having a large network of qualified institutions, did not show a high degree of attraction in either three-year period^17^.

This evidence indicates that assessing accessibility goes beyond simply considering the number and type of services provided in each region. Other variables must be considered. An essential aspect is the monitoring by the responsible bodies of compliance by qualified services with the reference parameters for regional planning and for the spatial distribution of these units, as recommended by the Ministry of Health^2^.

The displacement of patients for the treatment of stomach and colorectal tumors remained stable in the two three-year periods analyzed. The scarcity of investigations into geographical accessibility in the treatment of stomach cancer in Brazil limits comparison between studies and hinders more in-depth analysis. A Canadian study identified an association between distance from health regions to radiotherapy centers and colorectal cancer mortality, regardless of sociodemographic factors^21^. In the state of São Paulo, although displacement patterns have remained stable, there has been an increase in mortality from this type of cancer^1^.

The increase observed in the displacement of lung cancer patients for surgical and chemotherapy treatments may reflect barriers in access to care. A study carried out in Minas Gerais^22^ identified various obstacles to the diagnosis of this neoplasm, such as poor infrastructure, limitations in the public health network, lack of professional training, and the need for long journeys for treatment. In the state of São Paulo, there is probably a similar scenario, especially in regions with poor infrastructure.

Between the three-year periods analyzed, there was a reduction in the displacement of patients in the three therapeutic modalities related to cervical cancer. However, this decrease was not reflected in mortality. Between 2014 and 2020, there was an increase in mortality rates for this neoplasm^23^, suggesting that less mobility did not translate into better quality of care. Despite the expansion in the supply of services, the fragmentation of the SUS network remains a major obstacle, compromising the continuity of care throughout all stages of care. This disarticulation negatively affects the effectiveness of screening, the time taken for diagnostic confirmation after altered results and the speed with which treatment can be started^24^. These gaps in care help explain the difficulty in achieving significant results in controlling the disease, even in the face of the reduction observed in displacements.

Between the periods before and after the implementation of Oncology Regulation and RRAS, there was an increase in the number of patients diagnosed with breast cancer traveling to surgery. In Brazil, there is evidence that many women travel long distances to receive cancer treatment. Studies of SUS patients have shown that most of them must travel between 150 and 320 km from their homes to receive care^8,10^. This is a global reality: the greater the distance between the patient’s home and the place of treatment, the worse the prognosis. A study carried out in the United States highlighted the importance of geographical proximity to specialized centers for the appropriate management of early-stage breast cancer^25^.

In the case of prostate cancer, there was a reduction in the number of patients traveling for chemotherapy and radiotherapy between the three years analyzed. This decrease may be associated with a reduction in the need for these treatments. A study based on SUS data identified a drop in the availability of radiotherapy and chemotherapy for prostate cancer, concomitant with an increase in surgical procedures^26^.

Regarding the RRAS of attraction, no significant changes were observed in the regions of attraction of patients between the three years analyzed. These centers have a high capacity for care in various specialties, which explains the shift towards these regions. The concentration of highly complex services is justified by the need to specialize and gain scale, and the effect of distance is less decisive in these cases. However, in the context of primary health care and basic specialized services, territorial dispersion becomes fundamental, given that geographical accessibility has a strong correlation with the use of services^5^. We therefore recommend an oncology care planning model that combines the strategic concentration of highly complex services with the optimized distribution of primary health care and basic specialized services.

For health managers, it is essential to understand the demands for human and material resources in their region. Analysis of patient flows in the territory makes it possible to assess the supply of cancer services close to the areas of residence of the reference population, helping to optimize access to specialized treatment. In the long term, better geographical accessibility has consequences for critical determinants of therapeutic adherence, with a reduction in direct costs, such as transportation, accommodation, and meals for patients and companions, and mitigation of psychosocial costs^11^.

The second three-year period analyzed coincided with a regulatory change in Brazilian oncology: the definition of minimum annual parameters for surgical, radiotherapy, and chemotherapy procedures as a criterion for qualifying high-complexity services^2^. Although the impact of this change was not measured in this study, since the ordinance was published at the end of 2019, it is plausible to assume that this measure will contribute to reducing displacements by strengthening regional planning and consolidating the cancer diagnosis and treatment network.

Inequalities in accessibility to cancer treatment involve multiple aspects. It is important to note that the criteria for qualifying high-complexity oncology health facilities, according to Brazilian legislation^2^, consider factors such as the territory covered by care and its corresponding population, the general and specialized services offered by each hospital, the functioning of regional regulation mechanisms, and the minimum production of health actions. However, planning access to cancer services must be guided by social conditions and the real needs of the population, to reduce inequalities.

The sociodemographic profile of the patients remained stable between the periods analyzed, except for the education variable. The literature on geographical accessibility and sociodemographic factors is still scarce, which limits broader comparisons. In the case of schooling, the results of this study suggest that greater travel among individuals with a higher level of education may be associated with greater availability of financial resources for travel.

The distribution of tumors by staging remained stable between the three-year periods. However, the analysis by RRAS revealed variations in the movement of patients towards the centers of attraction and certain adjacent RRAS. Despite the recognized epidemiological relevance of sociodemographic and biological factors, it is likely that the changes observed in displacement patterns are more directly associated with transformations in the social determinants of health in the state of São Paulo. These determinants include increased access to health services and greater availability of complementary diagnostic tests. Moreover, it is important to consider the improvements in the social and economic conditions of part of the population, reflected in the increase in displacement among patients with higher levels of education in the second three-year period.

Brazilian legislation makes it compulsory for institutions qualified in high complexity oncology to keep and send data, regardless of whether they are public or private^2^. A limitation of this study is the failure to include cases from part of the oncology services in the RHC/SP, i.e. private institutions in the state that are not licensed by the SUS. This omission may restrict the generalization of the results to the entire population of São Paulo and to the full range of health establishments in the state. This omission may restrict the generalization of the results to the entire population of São Paulo and to the state’s health establishments. Another limitation of the study was the inclusion of only analytical cases - i.e. diagnosed and treated in the institution that registered the case - whose data was available for analysis. The exclusion of non-analytical cases, referring to patients who arrived at the institution with an established diagnosis, is due to the lack of information on where the previous procedures were carried out, which made it impossible to analyze the journeys made.

A central methodological contribution of this study is the analysis of patient flows between the RRAS, going beyond conventional approaches to assessing geographical accessibility. While previous studies have focused on the municipality as the unit of analysis and emphasized long-distance travel for treatment^8^, our RRAS-based approach offers a more comprehensive perspective by incorporating the principles of comprehensive care in the regional context of patients’ residence.

## CONCLUSION

The implementation of the RRAS and Oncology Regulation in the state of São Paulo has led to significant changes in the patterns of access to cancer treatment, with marked variations between regions, tumor types, and therapeutic modalities. There was stability in the distribution of referral centers between the periods analyzed, while the macro-regions showed heterogeneous dynamics: a reduction in displacements in the North and Northwest regions, and an increase in the South region. Regarding the type of tumor, three distinct patterns of displacement were identified: a reduction for cervical and prostate cases, stability for breast, stomach, and colorectal tumors, and a specific increase for lung cancer. It should be noted that in the case of cervical cancer, the decrease in displacements occurred in all therapeutic modalities. On the other hand, there was an increase in referrals for surgery in patients with lung cancer. These results highlight the importance of systematic monitoring of care flows and continuous qualification of regionalized planning in oncology, with attention to territorial and clinical specificities. This approach is essential for organizing the network, increasing its effectiveness and promoting greater equity in access to cancer treatment for the population of São Paulo.

## Data Availability

The data are not available.
