# Supplementary material for: Regionalization and regulation: impact on access to cancer treatment
Source: Rev Saude Publica. 2026 Mar 16;60:e9. doi: 10.11606/s1518-8787.2026060007257 (PMC12995285; doi:10.11606/s1518-8787.2026060007257)
Supplement: Translation

# Translation: Regionalização e regulação: impacto na acessibilidade ao tratamento de câncer

**Authors:** Luciane Simões Duarte, Mirian Matsura Shirassu, Adeylson Guimarães Ribeiro, Cristiane Murta-Nascimento, Marcela de Araújo Fagundes, Carolina Terra de Moraes Luizaga, Victor Wünsch

**Affiliations:** I Universidade de São Paulo. Faculdade de Saúde Pública. São Paulo, SP, Brasil; II Secretaria de Estado da Saúde de São Paulo. Centro de Vigilância Epidemiológica, Divisão de Doenças Crônicas Não Transmissíveis. São Paulo, SP, Brasil; III Fundação Oncocentro. São Paulo, SP, Brasil; IV Universidade Estadual Paulista. Faculdade de Medicina. Botucatu, SP, Brasil

**Keywords:** Oncologia, Regionalização da Saúde, Acessibilidade aos Serviços de Saúde, Hospitais Oncológicos

## Abstract

**OBJETIVO:**

Avaliar o impacto das Redes Regionais de Atenção à Saúde (RRAS) e da Regulação de Oncologia na acessibilidade geográfica ao tratamento oncológico dos principais tipos de câncer no estado de São Paulo.

**MÉTODOS:**

Estudo antes-depois, comparando os triênios anterior (2007–2009) e posterior (2017–2019) à implementação das políticas. Utilizaram-se dados do Registro Hospitalar de Câncer de São Paulo para analisar indicadores de deslocamento e atração de pacientes para cirurgia, radioterapia ou quimioterapia em seis tipos de câncer: estômago, colorretal, pulmão, mama feminina, colo do útero e próstata. As RRAS foram adotadas como unidades geográficas de análise, e os deslocamentos inter-RRAS foram categorizados em quartis (< 25%, 25%–49%, 50%–74% e ≥ 75%). Análise temporal foi realizada por mapas temáticos (QGIS, versão 3.28) e teste de qui-quadrado (α = 5%).

**RESULTADOS:**

Aproximadamente 25% dos pacientes se deslocaram para tratamento. As RRAS 6 (Capital), 9 (Bauru) e 13 (Ribeirão Preto) mantiveram-se como polos de atração. No Norte e Noroeste, as RRAS 11 e 12 apresentaram redução nos deslocamentos para todos os tipos de câncer; na RRAS 10, a queda ocorreu para tumores colorretal e pulmão. No Sudeste, as RRAS 3 e 5 não possuem serviços habilitados, exigindo deslocamentos, enquanto as RRAS 1, 2 e 4 mantiveram elevados percentuais. No Leste, as RRAS 14 a 17 registraram redução dos deslocamentos, exceto para câncer de pulmão. No Sul, os deslocamentos aumentaram nas RRAS 7 e 8. No geral, houve redução nos deslocamentos para câncer de colo do útero e próstata, e aumento para pulmão, especialmente cirurgia.

**CONCLUSÃO:**

A implementação das RRAS e da Regulação de Oncologia alterou a acessibilidade geográfica de forma heterogênea: houve melhora no Norte e Noroeste, piora no Sul, e estabilidade dos polos de referência. Destaca-se a necessidade de aperfeiçoamentos no planejamento regional, com atenção às desigualdades territoriais, ao tipo de tumor e à modalidade terapêutica.

## INTRODUÇÃO

Em 2023, foram estimados 125.200 novos casos de câncer no estado de São Paulo (excluindo câncer de pele não melanoma), sendo 51,3% em mulheres. Os tipos mais incidentes foram: mama, próstata, colorretal, traqueia, brônquio e pulmão, estômago e colo do útero^1^.

No Brasil, a assistência oncológica para diagnóstico e tratamento está organizada em três principais tipos de serviços especializados: as Unidades de Assistência de Alta Complexidade em Oncologia (Unacon), que atendem os tipos de câncer mais prevalentes; os Centros de Assistência de Alta Complexidade em Oncologia (Cacon), que oferecem tratamento para todos os tipos de câncer, exceto, em alguns casos, tumores raros ou pediátricos; e os hospitais gerais, que realizam cirurgias oncológicas e, quando necessário, encaminham pacientes para terapias complementares^2^.

O Ministério da Saúde implementou duas iniciativas com o objetivo de aprimorar a organização e o fluxo de pacientes oncológicos no Sistema Único de Saúde (SUS). A primeira, em 2008, foi a introdução da Regulação no SUS, com a finalidade de estruturar a oferta de serviços, garantir a equidade no acesso e assegurar uma assistência integral^3^. A segunda iniciativa, em 2010, foi a criação das Redes de Atenção à Saúde (RAS), concebidas para reduzir a fragmentação do sistema, ampliar a acessibilidade e integrar serviços de diferentes níveis de complexidade, com o suporte de sistemas logísticos e de gestão^4,5^.

No estado de São Paulo, essas políticas resultaram, em 2010, na criação da Central de Regulação de Ofertas de Serviços de Saúde (Cross) e, posteriormente, na implementação da Regulação de Oncologia^6^. Em 2012, foram instituídas as Redes Regionais de Atenção à Saúde (RRAS), com o objetivo de organizar os serviços de forma regionalizada e integrada^7^.

Apesar dos avanços na organização do sistema, a acessibilidade geográfica ao tratamento oncológico ainda representa um desafio relevante. Dados nacionais apontam que entre 49,2% e 60,7% dos pacientes com câncer precisam se deslocar de seus municípios de residência para receber atendimento especializado^8^. Essa situação resulta da centralização planejada dos serviços oncológicos, que não prevê a oferta de tratamento em todos os municípios. Embora essa diretriz tenha como objetivo garantir qualidade e integralidade na assistência, ela acarreta custos financeiros e emocionais adicionais aos pacientes e seus familiares^13^.

O conceito de acessibilidade vai além do simples acesso aos serviços de saúde, pois abrange tanto a entrada do usuário no sistema quanto a capacidade de oferta de atendimento e de resposta às necessidades de saúde da população. A acessibilidade pode ser analisada em duas dimensões: a sócio-organizacional, que abrange as características da oferta de serviços, excetuando-se aquelas de natureza geográfica, e a geográfica, que considera os aspectos espaciais que podem representar barreiras ao deslocamento do usuário. Esta última pode ser mensurada por meio de indicadores como: distância linear, tempo e custo de deslocamento, entre outros^14^.

Este estudo teve como objetivo avaliar o impacto das RRAS e da Regulação de Oncologia na acessibilidade geográfica ao tratamento oncológico dos principais tipos de câncer no estado de São Paulo, contribuindo para preencher lacunas do conhecimento sobre a evolução da acessibilidade geográfica ao tratamento oncológico no SUS e os efeitos das políticas específicas implementadas na área.

## MÉTODOS

Este estudo do tipo antes-depois comparou dois períodos independentes de três anos: pré-intervenção (2007–2009), anterior à implementação das RRAS e da Regulação de Oncologia no estado de São Paulo, e pós-intervenção (2017–2019), após a consolidação dessas estratégias. A utilização de triênios reduz a influência de variações pontuais no deslocamento dos pacientes, contribuindo para a estabilidade dos indicadores.

### Caracterização da Área do Estudo

Apesar de ocupar apenas 2,9% do território brasileiro, o estado de São Paulo concentra 21,9% da população do país, ou seja, mais de 44 milhões de habitantes distribuídos em 645 municípios^15^. Nesta análise, as RRAS foram adotadas como unidades geográficas de referência. Embora essa regionalização ainda não estivesse em vigor no triênio 2007–2009, optou-se por utilizá-la nos dois períodos considerados, a fim de garantir a comparabilidade dos resultados. As RRAS não se limitam a divisões geográficas ou administrativas, mas refletem características epidemiológicas, demográficas, socioeconômicas e a organização da rede local de serviços de saúde. Em 2024, a Secretaria de Estado da Saúde de São Paulo promoveu uma reorganização, ampliando o número de RRAS para 18^16^. Neste trabalho, contudo, manteve-se a divisão em 17 RRAS, conforme a configuração vigente nos dois períodos analisados.

### Base de Dados

Foram utilizados dados do Registro Hospitalar de Câncer de São Paulo (RHC/SP), sistema estadual gerenciado pela Fundação Oncocentro de São Paulo, que reúne informações de casos atendidos em estabelecimentos habilitados em alta complexidade oncológica^17^.

Nos dois triênios analisados, 66 instituições registraram casos de câncer no RHC/SP, sendo 48 Unacon, 15 Cacon e três hospitais gerais. Embora o número total de serviços tenha permanecido estável entre os períodos, ocorreram alterações em duas RRAS: na RRAS 6, houve redução de seis para cinco Unacon; e na RRAS 8, aumento de dois para três Unacon.

### Critérios de Inclusão

Foram incluídos pacientes ≥ 20 anos, residentes no estado de São Paulo, com diagnóstico de câncer durante os períodos do estudo e de acordo com a Classificação Internacional de Doenças para Oncologia (CID-O-3)^18^ para câncer de estômago (C16), colorretal (C18 a C20), brônquio e pulmão (C34), mama feminina (C50), colo do útero (C53) ou próstata (C61), incluindo casos *in situ*, submetidos a pelo menos um dos tratamentos: cirurgia, radioterapia ou quimioterapia. A amostra final compreendeu 52.128 pacientes entre 2007 e 2009 e 69.750 pacientes entre 2017 e 2019.

### Variáveis de Estudo

A acessibilidade geográfica foi avaliada por dois indicadores adaptados de Dama et al.^19^:

- Deslocamento de pacientes:

$$\frac{\text{Número de pacientes da RRAS de residência que realizaram tratamento em outra RRAS}}{\text{Número total de pacientes da RRAS de residência que realizaram tratamento oncológico}X100}$$

- Atração de pacientes:

$$\frac{\text{Número de pacientes não residentes que realizaram tratamento na RRAS}}{\text{Número total de tratamentos realizados na RRAS}X100}$$

Foram analisados o triênio de diagnóstico, a localização primária do tumor, o estadiamento tumoral, conforme o sistema TNM^20^, e a modalidade terapêutica (cirurgia, radioterapia ou quimioterapia). Além disso, foram examinadas as características sociodemográficas dos pacientes, incluindo sexo (masculino ou feminino), escolaridade (analfabeto, ensino fundamental completo ou incompleto, ensino médio ou superior) e idade (em anos).

### Análise de Dados

Os deslocamentos inter-RRAS foram categorizados em quartis (< 25%, 25%–49%, 50%–74% e ≥ 75%), pela facilidade de interpretação e pela geração de evidências científicas mais acessíveis e intuitivas. A análise temporal foi realizada mediante mapas temáticos comparativos entre os períodos (QGIS, versão 3.28) e teste de qui-quadrado (α = 5%). O *software* R (versão 4.2.0) foi utilizado para os procedimentos de análise.

### Aspectos Éticos

O estudo foi aprovado pelo Comitê de Ética em Pesquisa (CAAE: 75425623.0.0000.5421), em conformidade com as diretrizes das Resoluções n^o^ 466/12 e n^o^ 510/16 do Conselho Nacional de Saúde.

## RESULTADOS

Entre os períodos de 2007–2009 e 2017–2019, observou-se aumento no número de registros no RHC/SP para todos os tipos de câncer analisados. O câncer colorretal apresentou o maior crescimento percentual (58,2%), passando de 8.159 para 12.906 casos. Em seguida, destacou-se o câncer de mama, com aumento de 46,3% (de 16.413 para 24.007). Os tumores de pulmão, colo do útero e próstata apresentaram aumentos próximos a 20,0% cada: de 4.748 para 5.787, de 5.755 para 6.845 e de 12.932 para 15.920 casos, respectivamente. O câncer de estômago teve a menor variação, com aumento de apenas 3,4%, (4.121 para 4.285).

Observou-se um perfil semelhante entre os pacientes que se deslocaram nos dois triênios analisados, com proporções próximas de homens e mulheres em deslocamento para tratamento oncológico (cerca de 25%) e idade média de 58 anos. Entre o primeiro e o segundo triênio, verificou-se redução no deslocamento de indivíduos sem escolaridade (de 30,8% para 24,4%) e aumento entre aqueles com ensino fundamental (de 19,0% para 21,0%). As frequências dos estádios tumorais iniciais (TNM I e II) e avançados (TNM III e IV) por RRAS também foram semelhantes entre os períodos. Contudo, entre os indivíduos das RRAS 4, 6, 12, 14 e 16 com tumores em estádios iniciais, observou-se aumento no deslocamento, enquanto as RRAS 13 e 15 apresentaram diminuição. Já entre os pacientes com tumores em estádios avançados, verificou-se redução do deslocamento nas RRAS 9, 13 e 15.

Cerca de um quarto dos pacientes oncológicos no estado de São Paulo precisaram se deslocar para fora de sua RRAS de origem para realizar tratamento. O câncer do colo do útero foi o único que apresentou redução global nos deslocamentos para todas as modalidades terapêuticas. O câncer de próstata registrou redução no deslocamento total. Já o câncer de pulmão teve aumento, sobretudo nas cirurgias (+13,6%). Os tumores de estômago, colorretal e de mama mantiveram deslocamentos totais estáveis (Tabela 1).

**Tabela 1:** Distribuição de pacientes oncológicos com deslocamento inter-RRAS para tratamento, por tipo de câncer, modalidade terapêutica e triênio. Estado de São Paulo, 2007–2009 e 2017–2019.

Tipo de câncer e tratamento	2007–2009		2017–2019		Variação percentual	p^a^
	n	%	n	%		
Estômago						
Cirurgia	858	28,8	726	29,7	+3,1	0,489
Radioterapia	260	29,2	168	26,4	-9,6	0,254
Quimioterapia	669	27,7	870	29,3	+5,8	0,228
Total	1.787	28,5	1.764	29,1	+2,4	0,411
Colorretal						
Cirurgia	1.606	25,0	2.228	24,4	-2,4	0,347
Radioterapia	598	26,1	796	28,2	+8,0	0,106
Quimioterapia	1.245	22,1	1.802	21,6	-2,3	0,498
Total	3.449	24,1	4.826	23,8	-1,2	0,535
Pulmão						
Cirurgia	234	21,4	328	24,3	+13,6	0,097
Radioterapia	592	28,4	647	27,2	-4,2	0,382
Quimioterapia	949	25,5	1.130	27,0	+5,9	< 0,001
Total	2.071	23,5	2.105	26,6	+11,5	< 0,001
Mama						
Cirurgia	3.338	24,9	4.697	26,3	+5,6	0,007
Radioterapia	2.189	25,7	2.704	24,5	-4,7	0,069
Quimioterapia	2.770	24,8	3.609	23,8	-4,0	0,078
Total	8.297	25,1	11.010	25,0	-0,3	0,841
Colo do útero						
Cirurgia	1.304	32,4	1.243	28,0	-13,6	< 0,001
Radioterapia	625	28,4	543	24,7	-13,0	0,005
Quimioterapia	359	24,3	548	23,5	-3,3	0,573
Total	2.288	29,7	2.334	26,0	-14,3	< 0,001
Próstata						
Cirurgia	2.044	32,9	2.309	34,0	+3,3	0,171
Radioterapia	1.081	23,1	1.092	19,0	-17,7	< 0,001
Quimioterapia	218	17,2	191	11,1	-35,5	< 0,001
Total	3.343	27,5	3.592	25,2	-9,1	< 0,001

RRAS: Redes Regionais de Atenção à Saúde.

^a^ Teste de qui-quadrado.

A Figura 1 apresenta as variações em quartis dos deslocamentos de pacientes entre RRAS, por tipo de câncer e triênios. O mapeamento revela padrões regionais distintos de mobilidade para tratamento oncológico. Na região Noroeste (RRAS 10, 11 e 12), observou-se redução nos deslocamentos para todos os tumores nas RRAS 11 e 12. As regiões Norte e Centro (RRAS 9 e 13) mantiveram baixos percentuais de deslocamento, apontando relativa autossuficiência no atendimento oncológico. Na região Leste (RRAS 14 a 17), observaram-se variações heterogêneas de deslocamento, a depender do tipo de câncer. Na região Sul (RRAS 7 e 8), os percentuais de deslocamento permaneceram estáveis, com exceção da RRAS 8. Na região Sudeste (RRAS 1 a 5), destacam-se as RRAS 3 e 5, que não possuem estabelecimentos habilitados em oncologia de alta complexidade, resultando em deslocamentos obrigatórios. Além disso, a RRAS 6 manteve baixos percentuais de deslocamento, assinalando boa capacidade instalada e resolutividade local.

**Figura 1:** Distribuição espacial das RRAS por quartil de deslocamento de pacientes, por tipo de câncer e triênio. Estado de São Paulo, 2007–2009 e 2017–2019.

A Figura 2 apresenta as variações nos deslocamentos de pacientes entre as RRAS, de acordo com a modalidade terapêutica. Na região Noroeste (RRAS 10, 11 e 12), foram observadas reduções nos deslocamentos por modalidade; enquanto no Sul do estado (RRAS 7 e 8), houve aumento nos deslocamentos. Na região Sudeste (RRAS 1 a 5), as RRAS mantiveram elevados percentuais de deslocamento, exceto a RRAS 2 que apresentou redução para todas as modalidades de terapêuticas.

**Figura 2:** Distribuição espacial das RRAS por quartil de deslocamento de pacientes, por modalidade terapêutica e triênio. Estado de São Paulo, 2007–2009 e 2017–2019.

A Tabela 2 apresenta as proporções de atração de pacientes por RRAS nos dois triênios. Três RRAS – 6, 9 e 13 – destacaram-se como polos de referência, recebendo mais de 25% dos casos provenientes de outras regiões, em todas as modalidades terapêuticas. A RRAS 6 abrange a capital, e as RRAS 9 e 13 reúnem municípios populosos com boa infraestrutura de saúde. Na RRAS 9 destacam-se Bauru, Botucatu e Jaú, e na RRAS 13, Ribeirão Preto, Franca, São Carlos, Araraquara e Barretos – todos com mais de 100 mil habitantes. Essas RRAS apresentaram tendências distintas na atração de pacientes. A RRAS 6 registrou crescimento no volume de casos referenciados de outras regiões, mas as RRAS 9 e 13 tiveram redução nesse fluxo.

**Tabela 2:** Proporção de atração de pacientes oncológicos entre as RRAS, por modalidade terapêutica e triênio. Estado de São Paulo, 2007–2009 e 2017–2019.

RRAS de atração	2007–2009			2017–2019		
	Cirurgia	Radioterapia	Quimioterapia	Cirurgia	Radioterapia	Quimioterapia
1	8,5	6,7	8,1	12,4	1,3	6,1
2	1,1	0,0	1,8	8,1	5,8	7,4
3	-	-	-	-	-	-
4	14,0	3,8	10,3	29,1	0,0	0,0
5	-	-	-	-	-	-
6	28,0	27,9	28,4	34,9	32,6	35,7
7	0,0	0,0	0,3	0,6	0,3	0,6
8	0,9	0,3	1,1	0,3	0,3	0,4
9	52,7	50,7	49,4	42,4	41,1	41,7
10	0,8	1,0	0,4	0,6	1,4	0,6
11	0,4	0,6	0,5	1,2	1,4	0,5
12	0,9	0,0	0,7	2,3	0,8	1,0
13	39,6	37,8	38,0	28,8	26,2	27,3
14	2,6	9,6	3,3	1,9	16,2	2,5
15	16,1	14,0	12,8	6,3	5,6	4,9
16	4,8	7,4	7,2	11,5	14,5	13,6
17	0,2	0,2	0,2	0,0	0,0	0,0

RRAS: Redes Regionais de Atenção à Saúde.

Embora atendam pacientes de todo o estado, a maioria dos casos atraídos por essas três RRAS permaneceu concentrada em regiões adjacentes, padrão observado em ambos os triênios (Tabela 3).

**Tabela 3:** Proporção de pacientes não residentes atendidos nas três principais RRAS de atração, por RRAS de origem, modalidade terapêutica e triênio. Estado de São Paulo, 2007–2009 e 2017–2019.

Tipo de tratamento realizado e RRAS de atração	2007–2009		2017–2019	
	RRAS de origem	% de pacientes da RRAS de origem	RRAS de origem	% de pacientes da RRAS de origem
RRAS de atração 6				
Cirurgia	2	23,5	5	22,9
	5	22,6	2	20,0
	1	19,9	1	18,6
Radioterapia	2	24,1	5	25,9
	5	23,1	1	20,2
	1	18,8	2	17,3
Quimioterapia	2	23,8	5	26,1
	5	23,5	2	19,3
	1	18,3	1	17,0
RRAS de atração 9				
Cirurgia	10	28,8	8	44,4
	8	25,1	10	33,5
	13	14,4	13	12,2
Radioterapia	10	29,4	8	49,1
	8	26,6	10	28,7
	14	13,2	13	11,2
Quimioterapia	10	31,7	8	45,1
	8	24,7	10	34,2
	14	14,5	13	9,0
RRAS de atração 13				
Cirurgia	12	80,1	12	69,6
	15	6,5	15	10,0
	11	2,5	8	3,9
Radioterapia	12	80,0	12	57,5
	15	6,9	15	11,6
	11	2,8	8	6,6
Quimioterapia	12	78,3	12	54,8
	15	7,7	15	12,7
	11	2,9	8	6,1

RRAS: Redes Regionais de Atenção à Saúde.

## DISCUSSÃO

Os resultados constataram mudanças significativas na acessibilidade geográfica aos serviços de oncologia no estado de São Paulo após a implementação das RRAS e da Regulação de Oncologia. Verificou-se aumento no deslocamento de pacientes com câncer de pulmão para fora de suas RRAS de residência em busca de tratamento, enquanto os casos de câncer do colo do útero e de próstata apresentaram redução no deslocamento.

As regiões Norte e Nordeste do estado apresentaram redução nos deslocamentos para todas as modalidades de tratamento oncológico. Em contraste, a região Sul registrou aumento nesses deslocamentos, especialmente para procedimentos cirúrgicos. Permaneceram elevados os percentuais de deslocamento das regiões limítrofes em direção à capital, evidenciando desigualdades na distribuição dos serviços de atenção oncológica na Região Metropolitana de São Paulo.

Os dados deste estudo mostram uma expressiva desigualdade no acesso à atenção oncológica no estado de São Paulo, refletindo desafios persistentes, mesmo após a organização das RRAS e da Regulação de Oncologia. Observa-se concentração de serviços em polos específicos, como a RRAS 6 (São Paulo – Capital) e a RRAS 13 (região de Barretos e Ribeirão Preto), que concentram grandes centros de referência com elevada capacidade de atração de pacientes. Em contrapartida, a escassez de serviços de alta complexidade em regiões como a RRAS 3 (Franco da Rocha) e a RRAS 5 (Rota dos Bandeirantes) obriga os residentes a se deslocarem para outros territórios em busca de atendimento.

O estudo também demonstrou que, mesmo em RRAS com serviços habilitados em oncologia, notadamente as RRAS 1 (Grande ABC), 2 (Alto Tietê), 4 (Mananciais), 8 (região de Sorocaba) e 10 (região de Marília), os percentuais de deslocamento populacional superaram a média estadual. As maiores vulnerabilidades na assistência oncológica no estado concentram-se nas regiões adjacentes à capital e nas porções sul e sudeste.

O deslocamento de pacientes oncológicos tem sido amplamente documentado por estudos realizados no Brasil. No estado do Rio de Janeiro, pesquisa baseada em análise de redes sociais evidenciou diferenças significativas nos fluxos de atendimento e nos deslocamentos de pacientes com câncer do aparelho digestivo, ressaltando a centralidade persistente da capital^11^. Em São Paulo, estudo realizado em uma Unacon localizada em São Bernardo do Campo identificou que 43% dos pacientes eram provenientes de outros estados e revelou inconsistências nos registros de endereço em 10% dos prontuários médicos^9^. Em âmbito nacional, análise de mais de 12 milhões de procedimentos oncológicos constatou que pacientes das regiões Norte e Centro-Oeste percorrem as maiores distâncias para tratamento em função da concentração de polos de atenção oncológica nas regiões Sudeste e Nordeste^12^. Tais disparidades se mantêm ao longo do tempo, evidenciando a necessidade de revisar a distribuição dos serviços especializados em oncologia no país.

Além da presença de serviços habilitados, é importante considerar a quantidade e o tipo de serviço oncológico, pois esses fatores permitem identificar os vazios assistenciais no estado. As RRAS 1, 2, 4, 8, 10, 11, 12, 16 e 17, embora possuam todas Unacon e, em alguns casos (RRAS 7, 10 e 12), também Cacon, apresentaram elevados percentuais de deslocamento. Por outro lado, como esperado, RRAS com grande número de serviços habilitados (6, 9 e 13) destacaram-se como polos de atração. Entretanto, as RRAS 14 e 15, apesar da ampla rede de instituições habilitadas, não apresentaram alto grau de atração em ambos triênios^17^.

Essas evidências indicam que a avaliação da acessibilidade extrapola a simples consideração da quantidade e do tipo de serviços habilitados em cada região. Outras variáveis devem ser consideradas. Um aspecto essencial é o monitoramento, pelos órgãos responsáveis, do cumprimento pelos serviços habilitados dos parâmetros de referência para o planejamento regional e para a distribuição espacial dessas unidades, conforme recomenda o Ministério da Saúde^2^.

O deslocamento de pacientes para o tratamento dos tumores de estômago e colorretal manteve-se estável nos dois triênios analisados. A escassez de investigações voltadas à acessibilidade geográfica no tratamento do câncer de estômago no Brasil limita a comparação entre estudos e dificulta análises mais aprofundadas. Estudo canadense identificou uma associação entre distância das regiões de saúde aos centros de radioterapia e a mortalidade por câncer colorretal, independentemente de fatores sociodemográficos^21^. No estado de São Paulo, embora os padrões de deslocamento tenham se mantido estáveis, observa-se aumento na mortalidade por esse tipo de câncer^1^.

O aumento observado no deslocamento de pacientes com câncer de pulmão para tratamentos cirúrgicos e quimioterápicos pode refletir barreiras no acesso ao cuidado. Estudo realizado em Minas Gerais^22^ identificou diversos obstáculos ao diagnóstico dessa neoplasia, como infraestrutura deficiente, limitações da rede pública, despreparo profissional e a necessidade de longos deslocamentos para tratamento. No estado de São Paulo, há, provavelmente, um cenário semelhante, especialmente em regiões com carência de infraestrutura.

Observou-se entre os triênios analisados redução no deslocamento de pacientes nas três modalidades terapêuticas relacionadas ao câncer do colo do útero. Entretanto, essa diminuição não se refletiu na mortalidade. Entre 2014 e 2020, houve aumento nas taxas de mortalidade por essa neoplasia^23^, sugerindo que a menor mobilidade não se traduziu em melhor qualidade do cuidado. Apesar da ampliação da oferta de serviços, a fragmentação da rede do SUS permanece como entrave importante, comprometendo a continuidade do cuidado ao longo de todas as fases assistenciais. Essa desarticulação afeta negativamente a efetividade do rastreamento, o tempo para confirmação diagnóstica após resultados alterados e a agilidade no início do tratamento^24^. Essas lacunas assistenciais ajudam a explicar a dificuldade em alcançar resultados significativos no controle da doença, mesmo diante da redução observada nos deslocamentos.

Entre os períodos anterior e posterior à implementação da Regulação da Oncologia e das RRAS, observou-se aumento no deslocamento de pacientes diagnosticadas com câncer de mama para a realização de cirurgia. No Brasil, há evidências de que muitas mulheres percorrem longas distâncias para receber tratamento oncológico. Estudos com pacientes do SUS demonstraram que grande parte deles precisa viajar de 150 a 320 km de suas residências para receber atendimento^8,10^. Essa é uma realidade global: quanto maior a distância entre o domicílio da paciente e o local de tratamento, pior o prognóstico. Um estudo realizado nos Estados Unidos destacou a importância da proximidade geográfica a centros especializados para o manejo adequado do câncer de mama em estágio inicial^25^.

No caso do câncer de próstata, observou-se redução no deslocamento de pacientes para a realização de quimioterapia e radioterapia entre os triênios analisados. Essa diminuição pode estar associada à redução na necessidade desses tratamentos. Estudo baseado em dados do SUS identificou queda na disponibilidade de radioterapia e quimioterapia para o câncer de próstata, concomitante ao aumento na realização de procedimentos cirúrgicos^26^.

Em relação às RRAS de atração, não foram observadas mudanças significativas nas regiões de atração de pacientes entre os triênios analisados. Esses centros apresentam alta capacidade de atendimento em diversas especialidades, o que explica o deslocamento em direção a essas regiões. A concentração de serviços de alta complexidade é justificável pela necessidade de especialização e ganho de escala, sendo o efeito da distância menos determinante nesses casos. No entanto, no âmbito da atenção primária à saúde e dos serviços especializados básicos, a dispersão territorial torna-se fundamental, dado que a acessibilidade geográfica apresenta forte correlação com a utilização dos serviços^5^. Recomenda-se, portanto, um modelo de planejamento da atenção oncológica que combine a concentração estratégica de serviços de alta complexidade, com a distribuição otimizada da atenção primária à saúde e serviços especializados básicos.

Para o gestor em saúde, é imprescindível compreender as demandas por recursos humanos e materiais de sua região. Análise dos fluxos de pacientes no território permite avaliar a oferta de serviços oncológicos nas proximidades das áreas de residência da população de referência, contribuindo para a otimização do acesso ao tratamento especializado. A longo prazo, melhor acessibilidade geográfica gera consequências sobre determinantes críticos da adesão terapêutica, com a redução de custos diretos, como transporte, estadia e alimentação para pacientes e acompanhantes, e a mitigação de custos psicossociais^11^.

O segundo triênio analisado coincidiu com uma mudança regulatória na oncologia brasileira: a definição de parâmetros mínimos anuais para procedimentos cirúrgicos, radioterápicos e quimioterápicos como critério de habilitação dos serviços de alta complexidade^2^. Embora o impacto dessa mudança não tenha sido mensurado neste estudo, uma vez que a portaria foi publicada no final de 2019, é plausível supor que essa medida contribua para reduzir os deslocamentos, ao fortalecer o planejamento regional e consolidar a rede de diagnóstico e tratamento do câncer.

As desigualdades na acessibilidade ao tratamento oncológico envolvem múltiplos aspectos. É importante destacar que os critérios de habilitação dos estabelecimentos de saúde de alta complexidade em oncologia, conforme a legislação brasileira^2^, consideram fatores como o território de cobertura assistencial e sua população correspondente, os serviços gerais e especializados oferecidos por cada hospital, o funcionamento dos mecanismos de regulação regional e a produção mínima de ações de saúde. Contudo, o planejamento do acesso aos serviços oncológicos deve ser orientado pelas condições sociais e pelas necessidades reais da população, de modo a reduzir as iniquidades.

O perfil sociodemográfico dos pacientes manteve-se estável entre os períodos analisados, com exceção da variável escolaridade. A literatura sobre acessibilidade geográfica e fatores sociodemográficos ainda é escassa, o que limita comparações mais amplas. No caso da escolaridade, os resultados deste estudo sugerem que o maior deslocamento entre indivíduos com nível educacional mais elevado pode estar associado à maior disponibilidade de recursos financeiros para a realização de viagens.

A distribuição dos tumores por estadiamento permaneceu estável entre os triênios. Entretanto, a análise por RRAS revelou variações nos deslocamentos de pacientes em direção aos polos de atração e determinadas RRAS adjacentes. Apesar da reconhecida relevância epidemiológica dos fatores sociodemográficos e biológicos, é provável que as mudanças observadas nos padrões de deslocamentos estejam mais diretamente associadas às transformações ocorridas nos determinantes sociais da saúde no estado de São Paulo. Entre esses determinantes, destacam-se a ampliação do acesso aos serviços de saúde e a maior disponibilidade de exames complementares para diagnóstico. Além disso, é importante considerar as melhorias nas condições sociais e econômicas de parte da população, refletidas no aumento do deslocamento entre pacientes com maior escolaridade no segundo triênio.

A legislação brasileira estabelece a obrigatoriedade para as instituições habilitadas em alta complexidade em oncologia, da manutenção e do envio de dados, independentemente de serem públicas ou privadas^2^. Uma limitação deste estudo é a não inclusão, no RHC/SP, de casos provenientes de parte dos serviços oncológicos, ou seja, das instituições privadas do estado não habilitadas pelo SUS. Essa lacuna pode restringir a generalização dos resultados para toda a população paulista e para o conjunto completo de estabelecimentos de saúde do estado. Essa lacuna pode restringir estender os resultados para toda a população paulista e para o conjunto completo de estabelecimentos de saúde do estado. Registra-se, ainda, como limitação do estudo, a inclusão apenas dos casos analíticos – isto é, diagnosticados e tratados na própria instituição que registrou o caso – cujos dados estavam disponíveis para análise. A exclusão dos casos não analíticos, referentes a pacientes que chegaram à instituição já com diagnóstico estabelecido, deve-se à ausência de informações sobre o local onde foram realizados os procedimentos anteriores, o que impossibilitou a análise dos deslocamentos realizados.

Uma contribuição metodológica central deste estudo é a análise dos fluxos de pacientes entre as RRAS, superando abordagens convencionais de avaliação da acessibilidade geográfica. Enquanto estudos anteriores concentraram-se no município como unidade de análise e enfatizaram deslocamentos de longa distância para tratamento^8^, nossa abordagem baseada nas RRAS oferece uma perspectiva mais abrangente, ao incorporar os princípios da integralidade do cuidado no contexto regional de residência dos pacientes.

## CONCLUSÃO

A implementação das RRAS e da Regulação de Oncologia no estado de São Paulo promoveu alterações significativas nos padrões de acesso ao tratamento oncológico, com variações marcantes entre regiões, tipos de tumor e modalidades terapêuticas. Observou-se estabilidade na distribuição dos polos de referência entre os períodos analisados, enquanto as macrorregiões apresentaram dinâmicas heterogêneas: redução nos deslocamentos nas regiões Norte e Noroeste, e aumento na região Sul. Em relação ao tipo de tumor, foram identificados três padrões distintos de deslocamento: redução para os casos de colo do útero e de próstata, estabilidade para tumores de mama, estômago e colorretal, e aumento específico para câncer de pulmão. Destaca-se que, no caso do câncer de colo do útero, a diminuição dos deslocamentos ocorreu em todas as modalidades terapêuticas. Por outro lado, observou-se incremento nos deslocamentos para cirurgia em pacientes com câncer de pulmão. Esses resultados destacam a importância do monitoramento sistemático dos fluxos assistenciais e da qualificação contínua do planejamento regionalizado em oncologia, com atenção às especificidades territoriais e clínicas. Essa abordagem é essencial para a organização da rede, ampliação de sua efetividade e promoção de maior equidade no acesso ao tratamento oncológico à população paulista.

## Data Availability

Os dados não estão disponíveis.
